# Effect of dietary zinc supplementation on the gastrointestinal microbiome and host gene expression in the *Shank3B^−/−^* mouse model of autism spectrum disorder

**DOI:** 10.3389/fmicb.2025.1607045

**Published:** 2025-08-12

**Authors:** Giselle C. Wong, Yewon Jung, Kevin Lee, Chantelle Fourie, Kim M. Handley, Johanna M. Montgomery, Michael W. Taylor

**Affiliations:** ^1^School of Biological Sciences, University of Auckland, Auckland, New Zealand; ^2^Centre for Brain Research, University of Auckland, Auckland, New Zealand; ^3^Department of Physiology, University of Auckland, Auckland, New Zealand

**Keywords:** autism spectrum disorder, mouse model, *Shank3*, gut microbiome, host gene expression, bacteria, fungi, zinc

## Abstract

Shank gene variants are implicated in ~1% of people with autism, and mice lacking *Shank3* exhibit autism-like behaviours. Zinc deficiency and gastrointestinal problems can be common among people with autism, and zinc is a key element required for SHANK protein function and gut development. In *Shank3B^−/−^* mice, a supplementary zinc diet reverses autism-like behaviours. We hypothesise that dietary zinc may alter the gut microbiome, potentially affecting the gut-microbiome-brain axis, which may contribute to changes in autism-like behaviours. To test this, four types of gastrointestinal samples (ileum, caecum, colon, faecal) were collected from *Shank3B^−/−^* wild-type and knock-out mice on either control or supplemented-zinc diets, enabling us to examine the influence of—and interactions between—dietary zinc, the gut microbiome, and ASD-linked host genotype. Cage, genotype, and zinc diet each contributed significantly to bacterial community variation (accounting for 12.8, 3.9, and 2.3% of the variation, respectively). Fungal diversity was significantly lower in *Shank3B^−/−^* compared with wild-type mice on the control zinc diet, with specific fungal biota signatures detected among gut locations. Host metabolic genes, which may be regulated by the gut microbiota, and host genes involved in antimicrobial interactions were more highly expressed in *Shank3B^−/−^* mice. Metagenomic analyses revealed differential abundance of bacterial fatty acid biosynthesis and transporters (including zinc transport and neurotransmitter receptors) among our experimental groups. Overall these suggested increased activity of, or a switch towards, metabolic and microbial-host interactions that may benefit both host and microbe, in the presence of zinc. This raises the potential of manipulating both dietary zinc and the gut microbiota itself to ameliorate ASD-related behaviours and associated gastrointestinal issues. These data broaden understanding of the gut microbiome in autism and pave the way towards potential microbial therapeutics for gastrointestinal problems in people with autism.

## 1 Introduction

Autism spectrum disorder (ASD) is a neurodevelopmental condition that is clinically diagnosed by persistent deficits in three areas of social communication and interaction plus at least two of four types of restricted repetitive behaviours (American Psychiatric Association, 2013). Recent data indicate that ASD prevalence may be as high as 1 in 54 people (Maenner et al., 2020). It is clear that genetics play an important role in ASD causation (Abrahams and Geschwind, 2008; Sanders et al., 2012), while the potential influence of environmental factors has also come under increased scrutiny (Grabrucker, 2012; Karimi et al., 2017). One group of genes implicated in ASD encodes for the SH3 and multiple ankyrin (SHANK) repeat domain proteins. The SHANK protein family represents a group of important synaptic proteins localised in the post-synaptic density of the excitatory glutamatergic synapses in the brain (Naisbitt et al., 1999; Vyas and Montgomery, 2016), and mutations in the *Shank3* gene are associated with ~1% of all ASD cases (Leblond et al., 2014). Animal models of ASD have been created with ASD-*Shank* mutations to facilitate study of ASD mechanisms (Jiang and Ehlers, 2013; Nithianantharajah et al., 2017), including the *Shank3B^−/−^* mutant mouse model (ex13-16) which displays ASD-like characteristics including repetitive behaviours (grooming), anxiety, and deficits in social interaction (Peca et al., 2011). The *Shank3B^−/−^* knockout (KO) mouse model enables exploration of the interplay between the gut, the gut microbiota, and ASD. Indeed, a recent study of the microbiota of *Shank3B^−/−^* mice concluded that there was an imbalance, or dysbiosis, within the gut bacterial community, but that probiotic treatment with *L. reuteri* ameliorated ASD-like behaviours (Tabouy et al., 2018). SHANK3 may also play a role in gut permeability changes in ASD, with *Shank3* KO mice exhibiting decreased expression of the tight junction protein ZO-1 in the gut (Wei et al., 2017).

The micronutrient zinc is required for many processes in the body, including gastrointestinal (GI) epithelial barrier function (Skrovanek et al., 2014), gut permeability (Sturniolo et al., 2002), and enzyme functionality (McCall et al., 2000). Dietary zinc is mainly absorbed in the small intestine, where intestinal mucosa metallothionein proteins, zip proteins, and zinc transporters control zinc homeostasis (Maret, 2000; Dufner-Beattie et al., 2003; Liuzzi et al., 2004). Dietary zinc supplementation also confers protection against colitis along with zinc-mediated anti-inflammatory effects, improving gastrointestinal barrier function (Sturniolo et al., 2001; Sturniolo et al., 2002; Foligne et al., 2020; Souffriau et al., 2020). Zinc deficiency has been reported in the ASD population (Faber et al., 2009; Grabrucker et al., 2011b; Yasuda et al., 2015), potentially due to or leading to compromised nutrient acquisition from an individual’s diet (Gebhard et al., 1983), diarrhoea, and malnutrition due to reduced integrity of the gut epithelial barrier (Wapnir, 2000), and increased susceptibility to gut pathogens.

Gastrointestinal disorders (ranging from severe constipation to diarrhoea) are common in people with ASD (Buie et al., 2010; Babinska et al., 2020). In at least some cases this may be related to a compromised intestinal epithelial barrier (“leaky gut”) through which dietary products and/or bacterial metabolites may pass to enter into the bloodstream (D'Eufemia et al., 1996; Horvath and Perman, 2002; de Magistris et al., 2010; Kushak et al., 2016). Once in the bloodstream, immune responses and direct interactions with the central nervous system may be elicited. This brings into focus a potential contributing role for the gut microbiota in ASD. The gut-microbiota-brain axis refers to the bidirectional communication between gut microbes and the brain (Rhee et al., 2009; Cryan and O'Mahony, 2011; O'Mahony et al., 2015; Cryan et al., 2019), and there is evidence from both humans (De Angelis et al., 2015; Strati et al., 2017) and animal models (Hsiao et al., 2013; Buffington et al., 2016) for a link between the gut microbiota and ASD. Buffington et al. (2016) were able to reverse ASD-associated behaviours and elicit changes in brain neuronal activity in a mouse model of autism by reintroducing the probiotic bacterium *Lactobacillus reuteri*. Numerous studies have reported distinct gut microbiotas (including both bacteria and fungi) in people with and without ASD, while others report no such difference (Gondalia et al., 2012; Son et al., 2015; Strati et al., 2017; Kang et al., 2018; Liu et al., 2019). Nonetheless, the malleability of the gut microbiota, through for example diet changes, probiotics, or even faecal microbiota transfers between individuals, makes this an attractive option to explore in terms of developing potential microbiota-based therapies for ASD and the associated GI issues.

The SAM domain of SHANK3 proteins contains a zinc-binding site, which requires zinc to create structural homomers that act as a scaffold in the post-synaptic density (Grabrucker et al., 2011a), and to build post-synaptic protein complexes (Arons et al., 2016). SHANK3 deficiencies have also been associated with leaky gut and changes to the gut microbiome in a mouse model of ASD (Sauer et al., 2019). Zinc deficiency and loss-of-function *Shank* gene mutations have implications for assembly, localisation, and neurotransmission at the synapse (Rubenstein and Merzenich, 2003). Neurophysiology studies have shown that zinc is responsible for stabilising SHANK3 at the post-synaptic density and regulating excitatory glutamatergic synaptic transmission (Arons et al., 2016; Tao-Cheng et al., 2016). Disruption of this zinc-sensitive signalling system has been observed in *Shank3* mutations related to ASD, which may impact the functionality and plasticity of synapses in the brain and potentially result in ASD-like behaviours (Grabrucker, 2014; Arons et al., 2016). Supplementation of zinc in the diet is beneficial in the *Shank3B^−/−^* KO mouse model of ASD, with 6 weeks of postnatal supplemented dietary zinc resulting in the reversal of ASD-like behaviours as well as changes in the strength and structure of glutamatergic synaptic transmission in the striatum (Fourie et al., 2018). Moreover, zinc supplementation in pregnant *Shank3B^−/−^* KO mice can prevent the development of ASD-related behaviours in the offspring (Vyas and Montgomery, 2016). Taken together, these studies show the importance of zinc-dependent signalling in not only regulating SHANK at the synapse but also how this manifests as ASD deficits at the behavioural level.

The importance of diet (Yap et al., 2021) and other potential confounders (Vujkovic-Cvijin et al., 2020) to ASD have recently been highlighted. Disparities among previous studies with regards to methodologies and different populations also raise the question of whether the *identities* of the microorganisms present is the most important factor to consider. Rather, the *function* of the gastrointestinal microbiome could potentially be more informative when investigating differences among cohort or experimental groups. Indeed, the prevalence of GI disorders among many on the autism spectrum (Coury et al., 2012; Babinska et al., 2020) indicates the potential contribution of a dysbiotic microbiome. How these gastrointestinal microbes interact with the host, and the mechanism(s) by which dietary zinc treatment influences gut inflammation, are yet to be determined.

The link between dietary zinc and ASD behaviour suggests that the gut-microbiota-brain axis is a target for zinc-dependent signalling pathways in ASD. Based on this, along with known influences of the gut microbiota on gut-brain communications (Hsiao et al., 2013), we hypothesised that the synaptic and behavioural changes observed with dietary zinc in *Shank3B^−/−^* KO mice are linked to changes in the gut microbiota. Employing a combination of amplicon, metagenomic and metatranscriptomic sequencing, we sought to determine the effect of dietary zinc and host genotype on (1) the gut microbiota composition (bacteria and fungi), (2) host gene expression in the gut, and (3) microbiome-level functional differences.

## 2 Materials and methods

### 2.1 Animals

This study was approved by the University of Auckland’s Animal Ethics Committee (AEC#1299 and AEC#1969) and experimental procedures were in adherence to the ARRIVE guidelines. Shank3^ex13–16−/−^ mice (B6.129-Shank3^tm2Gfng^/J), created by deleting exons 13–16 in the *Shank3* gene (Peca et al., 2011), were imported from Jackson Laboratories, Bar Harbor, ME, United States and housed in the Vernon Jansen Unit animal facility at the Faculty of Medical & Health Sciences, University of Auckland. Heterozygous x heterozygous breeding pairs were bred to generate wild type (WT), heterozygous and homozygous knockout (KO) offspring mice. WT and KO were genotyped by PCR as described previously [52]. After weaning (~20 days post-birth), wild-type *Shank3B*^+/+^ (WT) mice and *Shank3B^−/−^* knockout (KO) mice were randomly assigned to one of two dietary experimental groups: a control zinc diet [30 ppm; (Tallman and Taylor, 2003; Vyas and Montgomery, 2016; Fourie et al., 2018)] or a supplemented zinc diet (150 ppm), for 6 weeks (Research Diets, Brunswick, NJ, United States) (D19410B and D06041101, Supplementary Table 1). Diets were identical in composition except for the zinc level. Four experimental groups were examined: (1) WT30 (wildtype control mice fed 30 ppm zinc; *n* = 46); (2) WT150 (wildtype control mice fed 150 ppm zinc; *n* = 48); (3) KO30 (*Shank3B^−/−^* mice fed 30 ppm zinc; *n* = 38); (4) KO150 (*Shank3B^−/−^* mice fed 150 ppm zinc; *n* = 46). Knockout and wildtype mice of the same sex were housed together in individually ventilated cages (maximum of 6 mice/cage), with a 12:12 h light:dark cycle and food available *ad libitum*.

### 2.2 Sample collection and DNA/RNA extraction

After 6 weeks on the control or supplemental zinc diet, faecal samples from 9-week-old WT and *Shank3B^−/−^* KO mice were collected and immediately frozen at −20°C. Mice were euthanised by CO_2_ and cervical dislocation, then gut tissue samples (ileum, caecum, colon) were collected into tubes containing RNA*later* (Ambion, Inc.).

The Qiagen AllPrep DNA/RNA Mini Kit (Qiagen, United States) was used to extract DNA and RNA according to the manufacturer’s instructions, with minor alterations as follows. Firstly, bead beating was carried out using MP Biomedicals lysing matrix E bead tubes (containing 1.4 mm ceramic spheres, 0.1 mm silica spheres, and one 4 mm glass bead) in the Qiagen TissueLyser II at a frequency of 30 Hz for 2 min. Extracted RNA was treated with DNaseI (Life Technologies, Auckland, New Zealand) and checked for DNA contamination using long cycle PCR targeting the V3-V4 region of the 16S rRNA gene with the following conditions: 95°C for 3 min, 50 cycles consisting of 30 s at 95°C, 30 s at 55°C, and 30 s at 72°C, followed by 72°C for 5 min.

### 2.3 PCR and amplicon sequencing

The hypervariable V3-V4 region of bacterial 16S rRNA genes was amplified using primers 341F and 785R (Klindworth et al., 2013) synthesised with an Illumina adaptor for multiplex sequencing (Supplementary Table 2). The fungal Internal Transcribed Spacer 2 (ITS2) genomic region was amplified using the ITS4 and ITS3mix1–5 primers with an Illumina adaptor (Supplementary Table 1) (Hoggard et al., 2018). Paired-end sequencing generating 2 × 300 bp reads was conducted by Auckland Genomics Ltd. (University of Auckland, New Zealand) on an Illumina MiSeq with V3 chemistry.

### 2.4 RNA sequencing

Extracted RNA of intestinal tissue from a subset of mice (4 treatment groups × 2 gut locations (ileum, colon) × 3 replicates = 24 samples) was sent to Otago Genomics (University of Otago, New Zealand) for ribosomal depletion (Ovation Complete Prokaryotic with mouse AnyDeplete module add-in, Tecan, United States), generation of one equimolar library pool, and paired-end sequencing generating 2 × 125 bp reads, using four lanes of two rapid flow cells on an Illumina HiSeq 2500 with V4 chemistry.

### 2.5 Shotgun metagenome sequencing

A subset of faecal and ileum samples was selected for shotgun DNA metagenomic sequencing, matching the samples selected for RNA sequencing but with additional ileum samples due to low DNA concentration (3 faecal samples per experimental group; 6 ileum samples for WT150 & KO 30; 7 ileum samples for WT30 & KO150). Rubicon Thruplex DNA nano libraries were prepared to create 550 bp fragments for 2 × 125 bp paired-end sequencing using the Illumina HiSeq 2500 platform with V4 chemistry at Otago Genomics.

### 2.6 Bioinformatics and statistical analyses

#### 2.6.1 Bacterial amplicon analyses

Amplicon sequence variants (ASVs) were generated from the 16S rRNA gene paired-end sequence files using the DADA2 package [v1.14.0; (Callahan et al., 2016)] in the R environment [v3.6.1; (RCoreTeam, 2019)]. Sequences were quality filtered, dereplicated, and merged, with merged sequences of 400–430 bp retained. Chimaeras, singletons, and ASVs representing less than 0.00001% of total sequences were removed. The remaining ASVs were taxonomically assigned using the SILVA database [v132; (Quast et al., 2013)]. A rarefaction threshold of 8,000 reads/sample was applied, leading to the exclusion of two ileum samples and one colon sample from subsequent analyses. Permutational multivariate analysis of variance (PERMANOVA) with 999 permutations of the data using the ‘vegan’ package (Oksanen et al., 2019) was undertaken to determine significant sources of variation within the microbial data. Kruskal-Wallis and Dunn tests (Dinno, 2017), with false discovery rate-adjusted *p*-values to account for multiple pairwise comparisons, were used to identify differentially abundant taxa across experimental groups.

#### 2.6.2 Fungal amplicon analyses

Zero-radius operational taxonomic units (ZOTUs) with 100% ITS2 sequence similarity were generated using a customised pipeline with USEARCH [v11; (Edgar, 2010)]. After primer removal, forward reads were trimmed to a maximum length of 220 bp, filtered with a maximum expected error rate of 1 and singletons were removed. The unoise3 algorithm and sintax classifier (Edgar, 2016a,b) were used to assign taxonomy with the UNITE reference database [UNITE_22.08.2016; (Nilsson et al., 2019)]. The ZOTU tables were further refined by removing ZOTUs which did not reach a minimum 0.001% relative abundance threshold across the entire dataset (Edgar, 2017). The data were subsampled to a depth of 500 reads/sample.

#### 2.6.3 RNA sequencing analyses

RNA sequencing files were processed using sickle [v1.33; (Joshi, 2011)] and trimmomatic [v0.38; (Bolger et al., 2014)] to trim adaptor sequences and quality trim reads with a minimum Phred quality score of 30 and a minimum length of 80 bp. Bacterial, archaeal and eukaryotic ribosomal RNA reads were removed using SortMeRNA [v2.1; (Kopylova et al., 2012)]. The STAR aligner [v 2.7.0, (Dobin et al., 2013)] was used to align the remaining reads to the Ensembl 84 mouse reference genome (Mus_musculus. GRCm38). Reads were then quantified using the feature counts software (Liao et al., 2014) and analysed using R [v3.6.1; (RCoreTeam, 2019)]. The R package edgeR (Robinson et al., 2010) was used to identify differentially expressed genes, and specific gene analysis was carried out using transcripts per million (TPM) units and the KEGG database (Kanehisa and Goto, 2000).

#### 2.6.4 Shotgun metagenome analyses

Adaptor sequences were removed using trimmomatic [v0.38; (Bolger et al., 2014)] from the raw sequencing data. Reads were trimmed based on a minimum Phred score of 30 with sickle [v1.33; (Joshi, 2011)], and co-assembled using metaSPAdes [v3.11.0; (Nurk et al., 2017)] with *k-mer* values 41, 51, 61, 81, 101. The 58,151 (>2 kb) assembled scaffolds were binned to create metagenome-assembled genomes (MAGs) using MetaBAT [v2.13; (Kang et al., 2019)], MaxBin [v2.2.6; (Wu et al., 2016)] and CONCOCT (v1.1.0); then dereplicated using DASTool [v1.1.1; (Sieber et al., 2018)] and dRep [v2.3.2; (Olm et al., 2017)]. Bins were validated using VizBin [v1.0.0; (Laczny et al., 2015)], resulting in 124 bacterial bins with >75% completeness, determined via CheckM [v1.0.13; (Parks et al., 2015)]. Of these MAGs, 83 were nearly complete (>95% completeness) and 7 bins were estimated to be 100% complete. Prodigal [v2.6.3; (Hyatt et al., 2010)] was used to predict open reading frames and protein sequences. Protein-coding sequences were annotated using UniProt (UniProt, 2021), UniRef100 (Suzek et al., 2007) and KEGG Orthologous groups databases (Kanehisa and Goto, 2000; Kanehisa et al., 2016) using USEARCH [v9.0.2132; (Edgar, 2010); minimum of 50% identity and e-value cutoff of 0.00]. Additionally, TIGRFAM [v14.0; (Li et al., 2021)] and Pfam [v32.0; (Mistry et al., 2021)] using HMMER [v3.1b2; (Finn et al., 2011); e-value cutoff of 0.001] were used for annotation and the Genome Taxonomy Database Toolkit [GTDB-Tk v1.5.0; (Chaumeil et al., 2019)] was used to assign taxonomy. A phylogenetic tree was created by identifying markers in genomes and creating a representative protein alignment of the main alignment used by GTDB. This alignment was inserted into a guide tree and the most likely taxonomy for each genome calculated by the placement of the genome in the tree, and the length of its branch from nearest neighbours. Bootstrapping (100 bootstraps) to test evolutionary models with IQ-TREE [v1.6.12; (Minh et al., 2020; Letunic and Bork, 2021)] were carried out and the tree was visualised and annotated using iTOL [v5; (Letunic and Bork, 2021)].

#### 2.6.5 Metagenome mapping to MAGs

Trimmed DNA sequence reads were subjected to removal of mouse reads with STAR (Dobin et al., 2013). Reads were mapped to MAGs using Bowtie [v1.2.0; (Langmead et al., 2009)] (WT30: 14 samples, 7 faecal & 11 ileum; WT150: 11 samples, 5 faecal & 6 ileum; KO30: 11 samples, 6 faecal & 5 ileum; KO150: 13 samples, 7 faecal & 6 ileum) (Bushnell, 2014). Mapped reads were normalised to 50,000,000 (number of reads/mapped read size * 1,000,000).

## 3 Results

### 3.1 Influence of mouse genotype and dietary zinc supplementation on bacterial and fungal community composition and diversity

**Figure 1:**
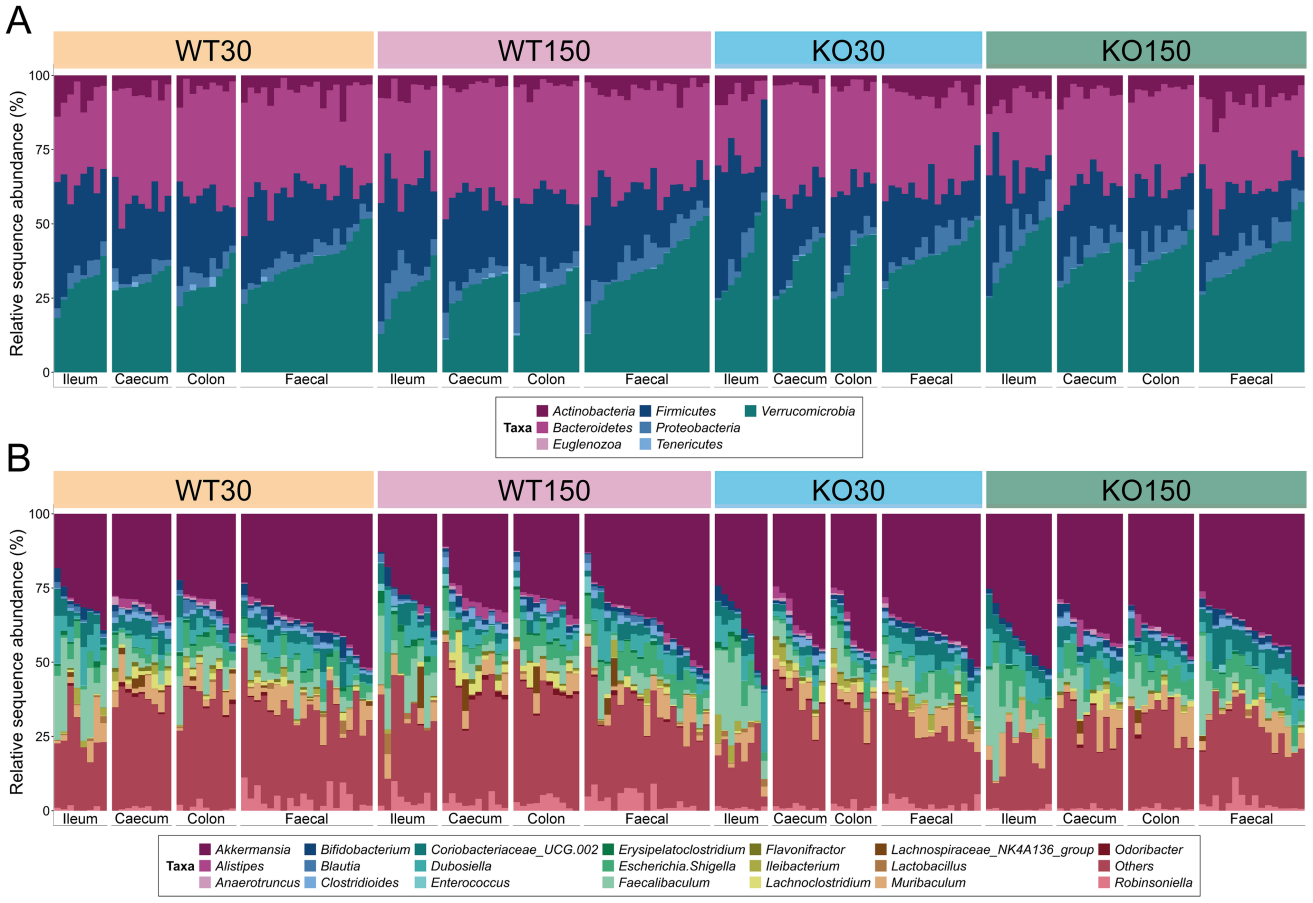
16S rRNA gene-based taxonomic summary plot of the relative abundance of bacterial phyla **(A)** and 20 most abundant genera **(B)** in each of the four experimental groups (wild-type control zinc diet WT30 *n* = 46, wild-type supplementary zinc diet WT150 *n* = 48, Shank3B^−/−^ KO control zinc diet KO30 *n* = 38, Shank3B^−/−^ KO supplementary zinc diet KO150 *n* = 46). All other genera and ‘unknown’ genus-level taxonomic assignments are grouped in ‘Others’. Gastrointestinal sections are noted as I (ileum), Ce (caecum), Co (colon), F (faecal).

Amplicon sequencing of 16S rRNA genes and ITS2 genomic regions was carried out to describe the bacterial and fungal communities, respectively. We observed that there was considerable overlap in bacterial (Figure 1) and fungal (Supplementary Figure S1) community compositions among the four experimental groups. The dominant bacterial phyla across the entire dataset were *Verrucomicrobia* (35.3 mean ± 7.8% S.D.), *Bacteroidetes* (31.9 ± 6.2%) and *Firmicutes* (22.2 ± 8.4%) (Figure 1A). The genus *Akkermansia* dominated across the entire dataset regardless of genotype or dietary zinc level (35.0 mean ± 8.1% S.D.), with *Faecalibaculum* (6.8 ± 6.5%), *Muribaculum* (4.4 ± 3.6%), *Escherichia*/*Shigella* (4.3 ± 2.6%) and *Dubosiella* (4.1 ± 3.8%) also abundant (Figure 1B).

Although the majority of detected fungal ZOTUs (67.3 mean ± 39.9% S.D.) could not be classified beyond kingdom level, the phylum *Ascomycota* was dominant among those taxa that could be assigned (20.1 ± 31.9%), with other ZOTUs classified to the phyla *Basidiomycota* (9.6 ± 25.7%) and *Zygomycota* (3.0 ± 8.1%) (Supplementary Figure S1). At genus level, the fungi *Rhodotorula* (6.4 ± 22.4%) and *Cyberlindnera* (6.2 ± 20.0%) were most prevalent among those identified, with nine other genera ranging from 0.01–2.70% relative abundance across the data set (Supplementary Figure S1B).

**Figure 2:**
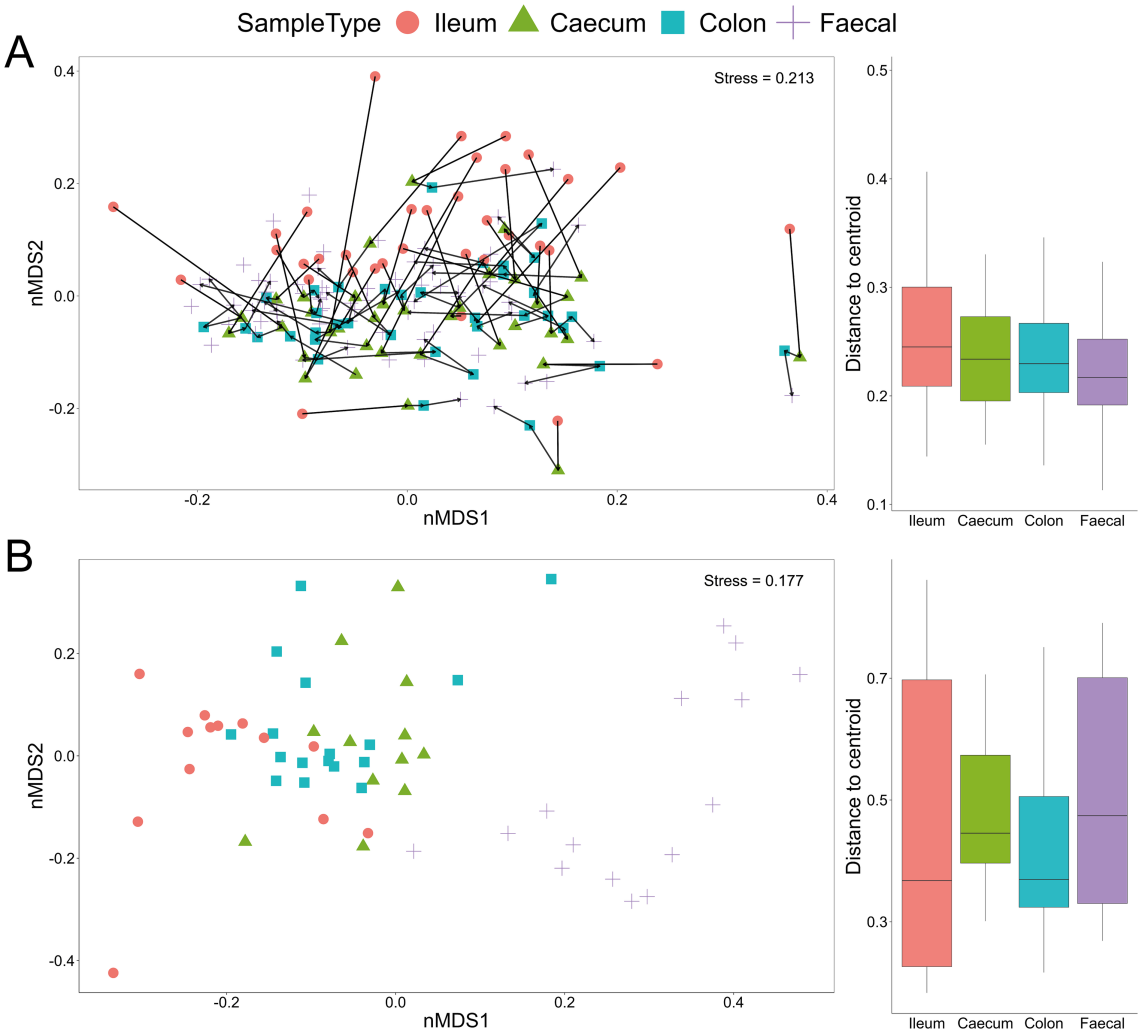
Beta-diversity Bray–Curtis dissimilarity non-metric multidimensional scaling (nMDS) plot from all samples with indicated sample type (ileum, caecum, colon and faecal) based on bacterial **(A)** and fungal **(B)** communities. Each line in **(A)** links the four GI samples for an individual mouse. Box and whisker plots show the distance from the centroid for the different sample types.

The lack of distinct microbial (bacterial and fungal) signatures among the four experimental groups, as shown by the taxonomic bar charts, was also evident in the non-metric multidimensional scaling plots for both bacteria and fungi (Figures 2A,B). Nevertheless, there were marked differences among communities from different gut sections, with the fungal communities notably differing along the gastrointestinal tract and even more so between gut sections and faecal samples (Figure 2B). Additionally, bacterial communities associated with ileum samples were overall less similar compared to those from the caecum, colon and faecal samples within an individual mouse, indicating a longitudinal shift in community compositions (Figure 2A).

**Figure 3:**
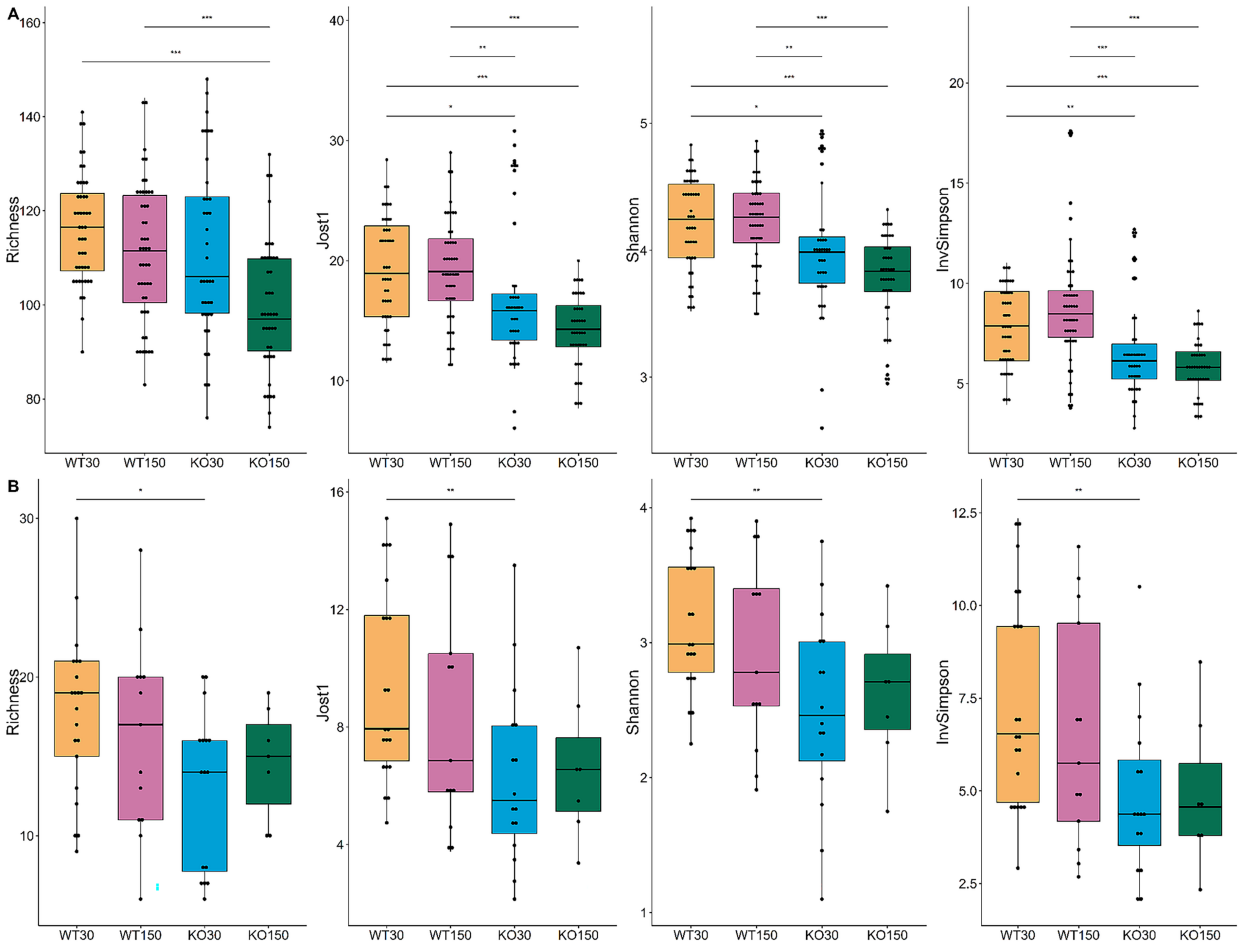
Alpha-diversity box and whisker plots based on Richness (observed taxa), Jost1, Shannon and Inverse Simpson index for bacteria **(A)** with WT30 *n* = 46, WT150 *n* = 47, KO30 *n* = 38, KO150 *n* = 43, and fungi **(B)** WT30 *n* = 21, WT150 *n* = 13, KO30 *n* = 16, KO150 *n* = 7. Statistical testing was carried out using the Kruskal-Wallis rank sum test with Wilcoxon test (3 degrees of freedom); ^*^p ≤ 0.05, ^**^p ≤ 0.01, ^***^p ≤ 0.001.

Bacterial community richness and diversity varied among samples, with 74 to 148 distinct bacterial ASVs observed per sample (Figure 3A). When aggregating samples from all gut locations for a given experimental group, there was significantly higher (*p* < 0.05) bacterial diversity in the WT compared to the *Shank3B^−/−^* KO mice (Figure 3A), irrespective of alpha-diversity metric and zinc diet. For the fungal ITS2 data, significantly lower diversity—supported by all four alpha-diversity metrics—was observed in the *Shank3B^−/−^* KO mice fed 30 ppm zinc compared to the wild-type mice fed the same amount of zinc (WT30) (Figure 3B), showing that gut fungal diversity was significantly decreased in *Shank3B*^*−/*−^ KO mice. However, this difference in fungal diversity was no longer observed when *Shank3B^−/−^* KO mice were fed the supplemented zinc diet (KO150) and there was no significant difference in alpha diversity compared to WT30 control mice. This suggests that dietary zinc may aid in the increase of fungal diversity to levels observed in WT mice.

In this study, mice of different genotypes were cage housed together and fed one of the treatment diets (control or supplemented zinc), with each cage comprising members of one sex. Based on bacterial 16S rRNA gene amplicon data, individual cages contributed the greatest amount (12.8%) of microbiota compositional variation (*p =* 0.001; Table 1). Gut sample location, mouse genotype, dietary zinc level and the interaction between genotype and zinc diet were also significant (*p* ≤ 0.008), explaining 11.4, 3.9, 2.3 and 1.1% of microbiota variation, respectively. A broadly similar trend was observed for the fungal data, whereby dietary zinc levels explained 3.7% of observed variation overall (*p* = 0.007), while gut sample location accounted for almost one-quarter (23.4%, *p* = 0.001) of variation (Table 2; Figure 2B). These results reflect substantially greater discrimination of fungal communities based on gut location than was observed for bacteria.

**Table 1:** PERMANOVA showing factors influencing 16S rRNA gene-based bacterial ASV composition, with 999 permutations.

Source	Df	SumsOfSqs	MeanSqs	F.Model	R^2^	Pr(>F)
Cage	1	1.5843	1.5844	31.8620	0.1284	0.0010
Sample type	3	1.4028	0.4676	9.4040	0.1137	0.0010
Genotype	1	0.4803	0.4803	9.6580	0.0389	0.0010
Zinc diet	1	0.2883	0.2883	5.7970	0.0234	0.0010
Genotype: Zinc diet	1	0.1324	0.1324	2.6630	0.0107	0.0080
Residuals	170	8.4533	0.0497	0.6850		
Total	177	12.3414	1.0000			

Sample type refers to sample location along the GI tract.

**Table 2:** PERMANOVA showing factors influencing fungal ZOTU composition, with 999 permutations.

Source	Df	SumsOfSqs	MeanSqs	F.Model	R^2^	Pr(>F)
Zinc diet	1	0.6988	0.6988	2.6457	0.03709	0.007
Sample type	3	4.4062	1.4687	5.5609	0.23389	0.001
Residuals	52	13.7340	0.2641		0.72902	
Total	56	18.8390			1.0000	

Sample type refers to sample location along the GI tract.

### 3.2 Specific microbial taxa as biomarkers for experimental groups

We identified specific microbial taxa that were differentially abundant among the four experimental groups, using Kruskal-Wallis rank-sum and Dunn tests with adjustments for multiple pairwise comparisons. A total of 74 bacterial ASVs differed significantly in abundance between the WT30 controls and the KO30 groups (Supplementary Table 2), with the majority classified as members of the phyla *Bacteroidetes* and *Firmicutes*, and the families *Muribaculaceae* and *Lachnospiraceae*. Individual ASVs with the lowest *p*-values, indicative of between-treatment differences (*p* < 3.78 × 10^−9^) were ASVs 182/155/135 (all *Akkermansia*), ASV 90 (*Lachnoclostridium*) and ASV 69 (*Muribaculaceae*). Sixty-five ASVs differed significantly (*p* < 0.05) in relative abundance between the KO30 and KO150 groups, and 65 ASVs also differed between the WT30 and KO150 groups, including the aforementioned bacterial taxa (Supplementary Table 2). Across all experimental groups, 24 fungal ZOTUs were identified as being differentially abundant (Supplementary Table 3). Only five of these ZOTUs could be classified below kingdom level: *Rhizopus* ZOTUs 5, 7 and 502, and *Aspergillus* ZOTUs 11 and 19 (Supplementary Table 3).

### 3.3 Effect of mouse genotype and dietary zinc supplementation on host gene expression

**Figure 4:**
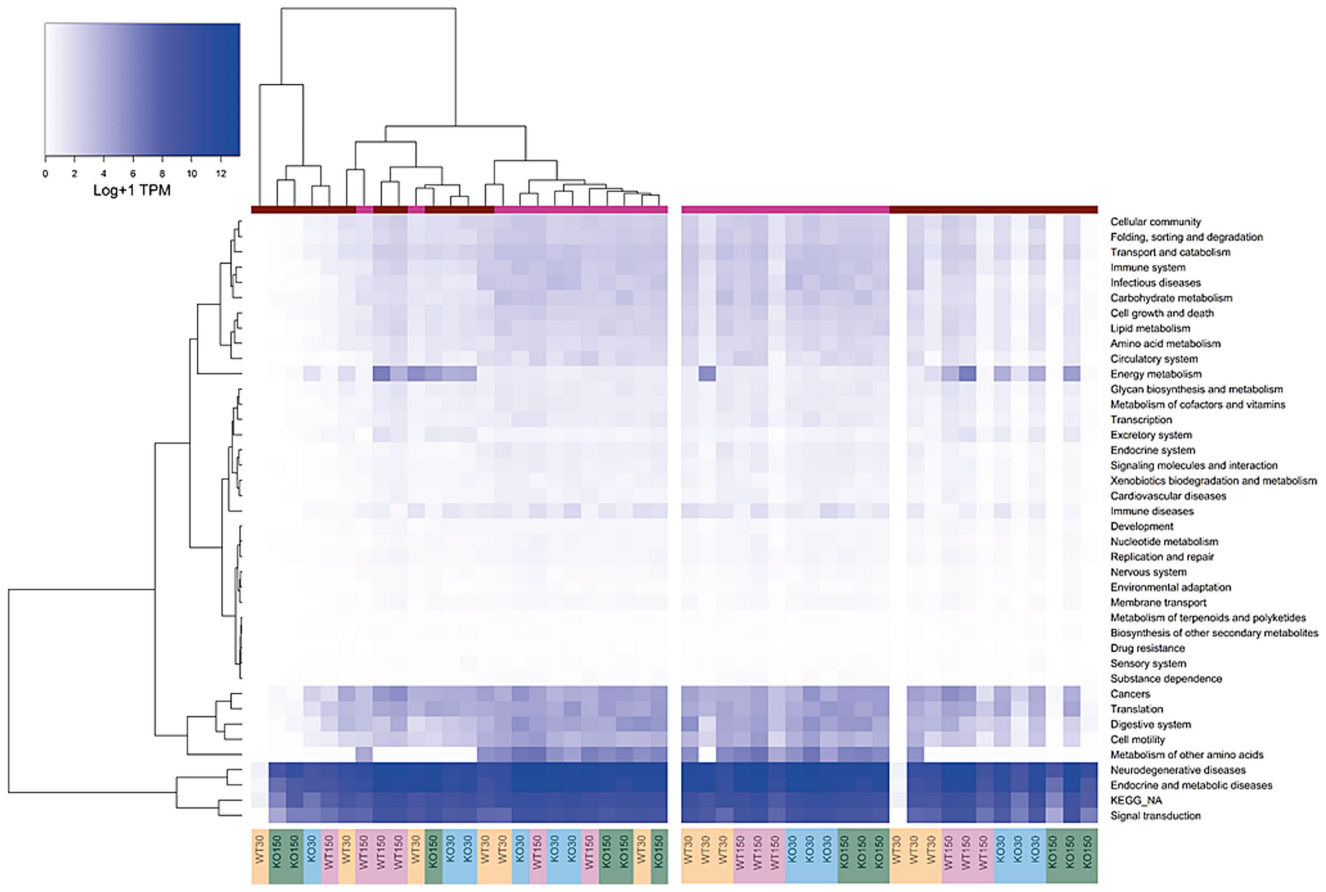
Heatmap of KEGG orthologous groups (level 2) expressed by the host (mouse) across the four experimental groups and two sample types (ileum and colon). RNA-seq data were normalised to total counts and presented as log +1 transcripts per million. Sample types are represented by the coloured bar at the top (pink = ileum, red = colon). Samples are re-ordered to allow clustering in the left panel, whereas the same samples without ordering are shown in the right-hand panel.

Differences in host gene expression were observed between gut sections and treatment groups. Host gene expression patterns correlated strongly with gut section (colon versus ileum). Compared to the colon, ileal samples exhibited lower expression of functional gene groups related to the digestive system, cell motility, and metabolism of amino acids, while genes relating to energy metabolism were typically more highly expressed in the colon (Figure 4). KEGG functional groups of genes related to signal transduction, endocrine and metabolic diseases, and neurodegenerative diseases were also highly expressed in all colon and ileum samples, encompassing ~ 86–89% of all mouse-associated transcripts (KEGG Level 2, Figure 4).

There were differences in KEGG functional groups associated with host gene expression between the WT and *Shank3B*^*−/*−^ KO genotypes as well as between the zinc diets within a genotype, with differences most notable among colon samples. Genes involved in energy metabolism were enriched in the colon of *Shank3B*^*−/*−^ KO mice (on both zinc diets) compared to wild-type mice on the control zinc diet. Additionally, there was decreased expression of immune disease genes on the supplemented zinc diet (150 ppm) in both KO and WT mice compared to the control zinc diet (30 ppm) in the colons of mice with these genotypes (Figure 4).

**Figure 5:**
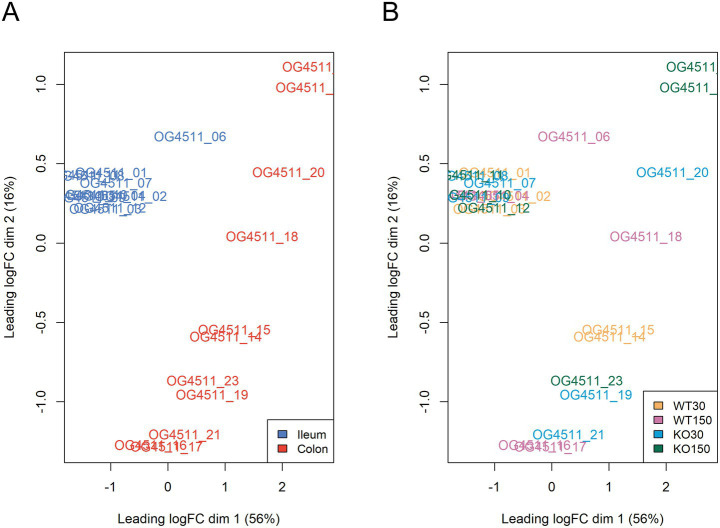
Multi-dimensional scaling plots of RNA-seq data, with distances showing leading logFC (the average of the largest absolute log fold changes between each pair of samples) coloured by sample type **(A)** and experimental group **(B)**.

Across the mouse host-associated data set, a total of 1,387 genes were significantly differentially expressed. Consistent with the clustering shown in Figure 4, ileum and colon samples were clearly separated in two-dimensional space (Figure 5A), with greater variability in the expression of these genes in the colon. There were 1,271 genes among the ileum samples and 741 genes among the colon samples which were significantly differentially expressed. Many of these were related to antimicrobial interactions (alpha-defensins, mucin 2, carbonic anhydrase 1, intelectin) and host metabolism genes that may be regulated by the gut microbiota (e.g., NADH dehydrogenases). By contrast, distribution of samples in the ordination did not correlate with experimental group, which encompasses zinc diet and mouse genotype (Figure 5B).

**Figure 6:**
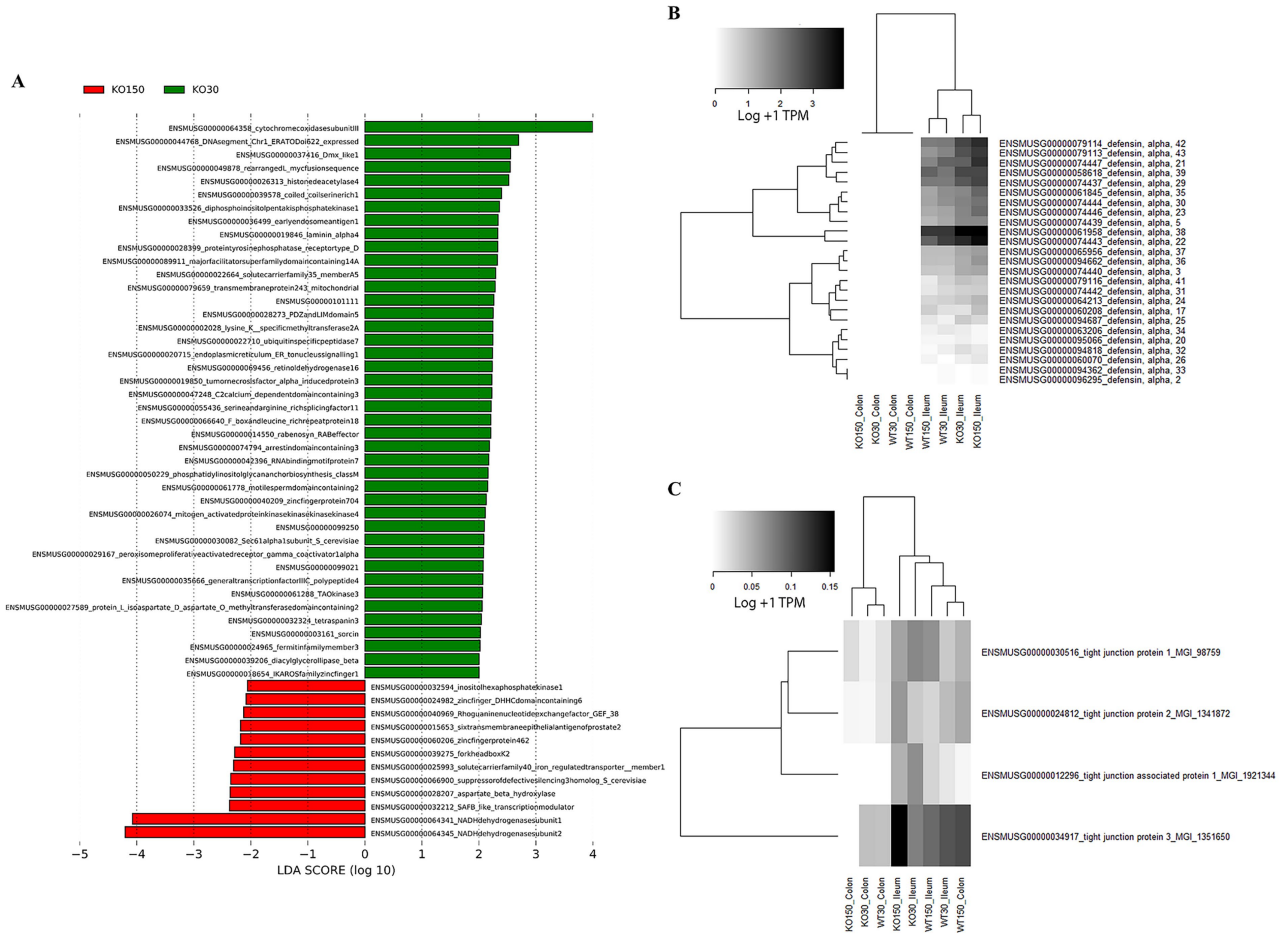
RNA-seq analyses of host (mouse) gene expression: **(A)** LEfSe analysis (with Kruskal-Wallis and Wilcoxon testing both set at *p* < 0.05, with the strategy all against all) identifying biomarker differences between KO30 vs. KO150 for both ileal and colon samples combined. Heatmaps of **(B)** alpha defensin genes, and **(C)** tight junction protein genes across the four experimental groups and two sample types. RNA-seq data were normalised to total counts and presented as log +1 transcripts per million. Samples within an experimental group and sample type were pooled together for analysis as biological replicates.

Therefore, to further investigate differences between experimental groups, linear discriminant analysis of effect size (LEfSe) was used with host and microbiome gene transcript data. This identified 54 genes (biomarkers) that were significantly differentially expressed between the KO30 and KO150 groups (Figure 6A). NADH dehydrogenases, DNA/RNA binding proteins, zinc finger and transmembrane proteins were among the most differentially expressed gene categories. We focused our subsequent analysis on two main categories: alpha defensins, due to their importance in the control of bacteria at the mucosal barrier, and genes related to tight junction proteins, which play an integral role in mediating gastrointestinal permeability (Chelakkot et al., 2018). Overall, alpha defensin genes were more highly expressed in the ilea of *Shank3B^−/−^* (KO) mice compared with the ilea of wild-type mice, with slightly higher expression in the supplemented zinc diet KO mice than without supplementation (Figure 6B). Expression of tight junction genes was significantly and markedly lower in the colon samples from *Shank3B^−/−^* KO mice receiving zinc supplementation compared to their wild-type counterparts on the same diet (*p* = 0.0139), while the ileum samples showed the opposite trend, and these ilea genes were overall more highly expressed than those in the colon (Figure 6C).

### 3.4 Genomic reconstructions of mouse gut bacteria, including previously unobserved taxa

**Figure 7:**
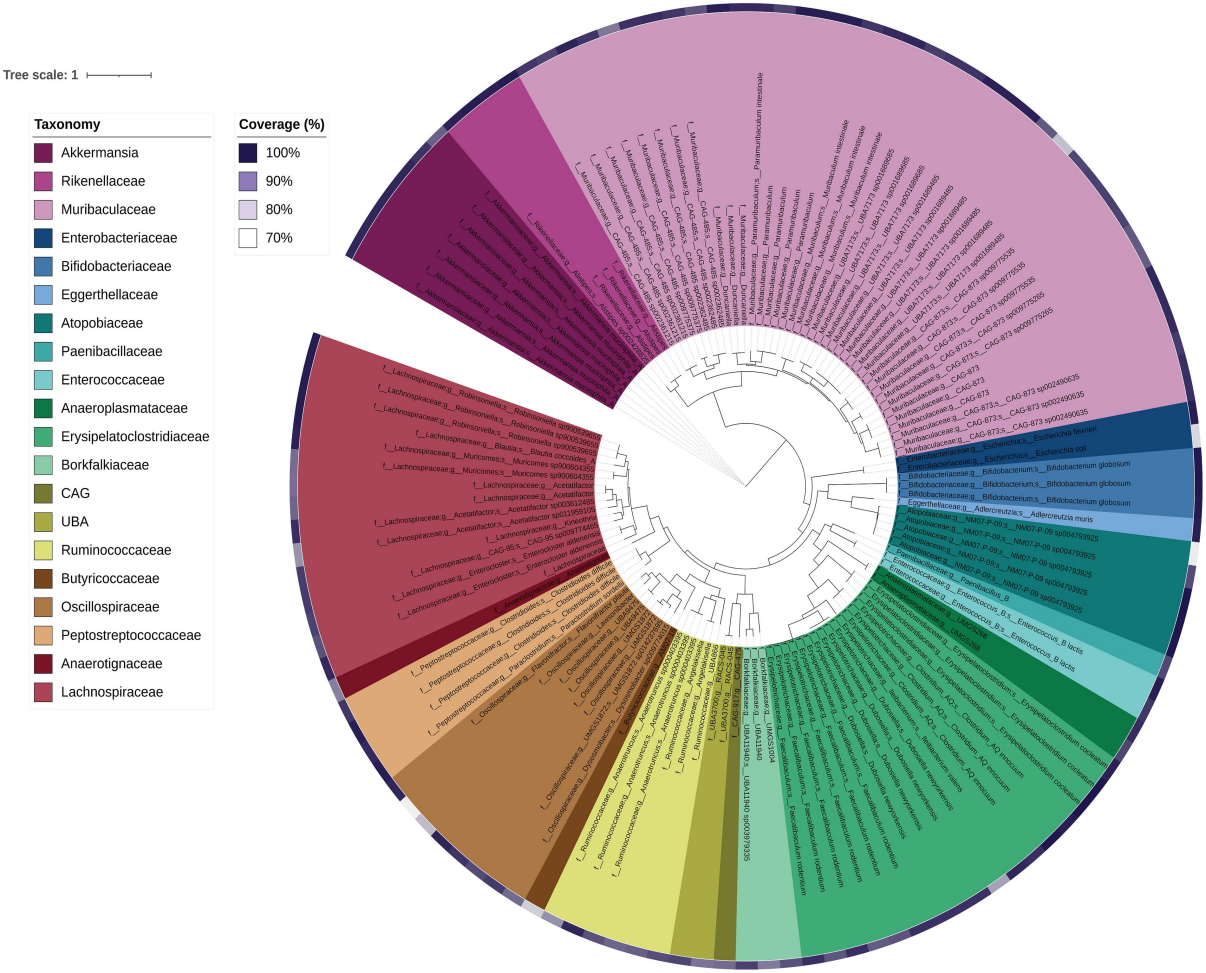
Phylogenetic tree of each MAG based on concatenated core genes visualised using iTOL. The colours represent different family groups and genome completeness is indicated by the purple colour gradient outside of the main tree.

DNA shotgun sequencing data were reconstructed into 124 metagenome-assembled genomes (MAGs). Seven were 100% complete (with 0–0.94% contamination), 104 were 90–99.78% complete (0–9.68% contamination), and 13 were 76.62–89% complete (0–14.75% contamination). Of these, 124 MAGs with ≤5% contamination and at least 70% estimated completeness were analysed further (Figure 7). Overall the majority of the MAGs were assigned to the families *Muribaculaceae*, *Lachnospiraceae* and *Erysipelatoclostridiaceae* (including *Faecalibaculum* and *Clostridium* genera), which was largely consistent with the faecal 16S rRNA gene amplicon data. As expected, general positive gut health indicator taxa *Akkermansia* and *Bifidobacteriaceae* were also present in the MAGs (Figure 7). MAGs were also obtained for members of the *Atopobiaceae*, *Eggerthellaceae*, *Borkfalkiaceae*, *Oscillospiraceae* and *Anaerotignaceae*, despite their not previously being detected in *Shank3^−/−^* mice bacterial amplicon data (Figure 1). The most highly abundant MAGs belonged to collections of *Akkermansia muciniphila* followed by *Paramuribaculum* (including *P. intestinale*) in faecal samples. In the ileum samples, MAG 22 (*Muricomes* sp900604355) was highly represented in WT30, WT150 and KO150 groups compared to the KO30 group. This same trend was also observed for MAGs representing *Akkermansia*, *Porphyromonas* bacterium UBA7173 and *Faecalibaculum*. Overall, we observed increased abundances of *Akkermansia*, *Porphyromonas* and *Faecalibaculum* in bacterial communities from KO mice on the supplemented zinc diet.

### 3.5 Major functional differences between experimental groups in microbiome-encoded genes for transporters, zinc-related genes, carbohydrate metabolism, and fatty acid biosynthesis

Differences in bacterial community function were determined using KEGG database annotations of MAGs. In terms of functional capacity, the KO150 group on the supplemented zinc diet displayed a greater relative abundance of genes for metabolism, environmental information processing, and cellular processes than other groups. For example, cellular process gene relative abundances differed significantly between KO150 and the groups KO30 (*p* = 0.037) and WT30 (*p* = 0.00018), and were highest in the KO150 group. Significant genotype differences were observed relating to two of these categories, environmental processing (*p* = 0.015) and cellular processes (*p* = 0.039). Between the dietary zinc treatments, cellular processes (*p* = 0.00084) and environmental processing (*p* = 0.0000056) differed most significantly. Cellular process genes differed significantly between KO150 and the groups KO30 (*p* = 0.037) and WT30 (*p* = 0.00018), with the same trend for environmental processing genes (KO30 *p* = 0.000091; WT30 *p* = 0.000021)—i.e. relative abundances were again highest in the KO150 group. By contrast, the abundance of mucin genes was greater in WT mice compared to KO mice, with increased presence in faecal samples compared to ileum samples within all experimental groups.

While sample location did not significantly affect the distribution of genes involved in carbohydrate metabolism, genotype (*p =* 0.0046), zinc diet (*p =* 0.011) and the interaction between genotype and zinc diet (*p =* 0.021) all significantly affected the number of protein-coding sequences annotated with carbohydrate metabolism in the microbial community. Zinc-related genes, including zinc transporters, metallopeptidases, and zinc-binding cytidine monophosphate, were more abundant in the KO150 group compared to the KO30 group, especially in the ileum. Saturated and unsaturated fatty acid synthesis genes were found in 93 bacterial taxa, with *Akkermansia muciniphila*, *Muribaculum intestinale*, and *Alistipes finegoldii* representing the top three taxa across the entire dataset with these genes. In the ileum samples, normalised count data revealed a lower abundance of fatty acid-related genes in KO30 (total number of genes = 1,346) compared to WT30 (total = 2,371), but an increase in KO150 mice (total = 2,026) towards WT30 levels. This trend was also seen in the faecal samples, WT30 (total = 2,843); KO30 (total = 2,166); KO150 mice (total = 2,368). Interestingly, in the KO30 faecal samples and KO150 ileum samples, *A. finegoldii* was not among the top three taxa with respect to fatty acid synthesis genes, instead being replaced by *Bacteroides salanitronis* (identified from KO30 faeces) and *Lachnoclostridium* sp. YL32 (KO150 ileum sample). Genes related to synthesis of propionate were significantly different between zinc diets (*p =* 0.029) and the interaction between genotype and zinc diet (*p =* 0.033), but not between the genotypes; these genes were associated with *Escherichia coli* K-12 MG1655, *Desulfitobacterium dehalogenans* and *Flavonifractor plautii*. In KO30 mice, the main propionate-associated organism was *F. plautii*, while *Escherichia coli* K-12 MG1655 was the top bacterium identified that is associated with propionate production and transport in both WT30 and KO150 mice.

## 4 Discussion

**Figure 8:**
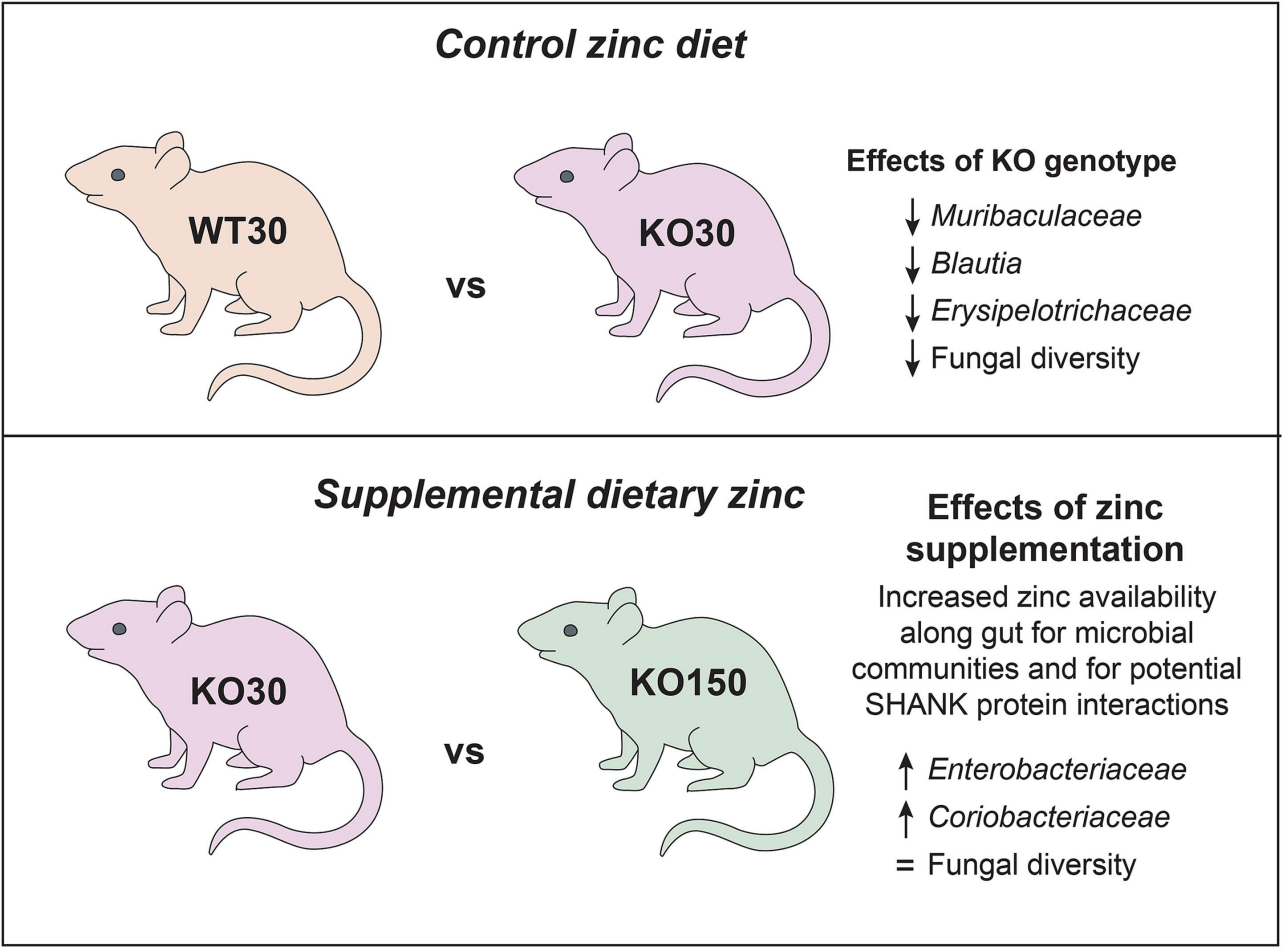
Summary of key microbiome findings from this study.

The role of the gut microbiota in ASD has come under considerable scrutiny in the past decade, with evidence for its importance coming from studies of both human populations and animal models (Hsiao et al., 2013; Buffington et al., 2016; Strati et al., 2017). Neural, immunological and hormonal pathways all offer potential mechanisms by which gut microbes may influence neurological development. Changes in gut microbial composition and resulting functional differences have been observed with differential dietary zinc intake in porcine and murine models as well as in human studies (Usama et al., 2018; Foligne et al., 2020; Pieper et al., 2020). We recently described dietary zinc-induced reversal of ASD behaviours in *Shank3B^−/−^* KO mice (Vyas and Montgomery, 2016; Fourie et al., 2018). Zinc plays a critical role in gut health and, together with published data linking the gut-microbiota-brain axis to ASD, these observations raise the intriguing possibility that the effects of dietary zinc on ASD could be linked to changes in the gut microbiota. In this study, we therefore examined the effects of dietary zinc and host genotype on (1) the gut microbiota in *Shank3B^−/−^* KO mice, (2) host gene expression in the gut, and (3) microbiome-level functional differences. Our major findings are summarised in Figure 8.

### 4.1 Zinc supplementation and host genotype influence microbiota composition

When considering the gut-associated microbiota, differences among individual mice were substantial. Even within a given dietary zinc level or genotype group, phylum-and genus-level bacterial community compositions varied greatly, albeit with overall dominance by certain taxa, most notably the genus *Akkermansia* (phylum *Verrucomicrobia*). Despite such inter-individual variation, which is consistent with previous autism gut microbiota studies (Bundgaard-Nielsen et al., 2020), our data reveal significant effects of host genotype, dietary zinc level, and their interaction. Specifically, for both bacterial and fungal data, across all alpha-diversity indices WT mice displayed significantly increased diversity compared with *Shank3B^−/−^* KO mice. This trend of decreased fungal and bacterial alpha-diversity in *Shank3B^−/−^* KO mice regardless of dietary zinc treatment suggests that host-associated gut microbes may be highly regulated by ASD-linked genetic variations occurring in key synaptic proteins in the central nervous system which can ultimately affect immunological interplay between host and microbe.

*Akkermansia muciniphila* is a mucin-degrading gut bacterium that improves gut barrier function by stimulating host mucous production and turnover, ultimately reducing GI tract inflammation via regulation of antimicrobial peptides in the gut stimulating host Toll-like receptors that are integral to host immunity (Garcia-Vello et al., 2024). In our study, *Akkermansia* was one of the most dominant bacterial taxa for all experimental groups and was also widely represented in the shotgun metagenomic data. Decreases in *A. muciniphila* have been observed in ASD, irritable bowel syndrome and Crohn’s disease, leading to the development of probiotic treatments with this microbe for these gut disorders (Gu et al., 2021).

The clearest differences related to dietary zinc levels and/or genotype were evident not at the level of entire bacterial communities, but rather at the level of specific ASVs, consistent with other gut microbiome studies (de Theije et al., 2014; Chen et al., 2017). For example, 74 ASVs, mostly from the families *Muribaculaceae* and *Lachnospiraceae*, were significantly differentially represented between the WT and KO groups that were both fed 30 ppm zinc. When comparing the two genotypes on the control zinc diet, *Muribaculaceae*, *Blautia* and *Erysipelotrichaceae* were more abundant in WT30 mice than KO30 mice. *Blautia* produces butyrate, a short-chain fatty acid (SCFA), and is considered beneficial for host health. Butyrate has positive anti-inflammatory effects at the mucosa and fortifies the epithelial barrier, with implications for the gut-microbiota-brain axis as it is able to cross the blood–brain barrier and produce neuromodulatory effects (Canani et al., 2011; Oleskin and Shenderov, 2016; Silva et al., 2020). Other SCFAs such as acetate, propionate and succinate can also be produced by members of the family *Muribaculaceae*. This family represents one of the largest proportions of gut bacteria found in mice, and has been suggested to play a similar role to the genus *Bacteroides* in humans, occupying similar niches and metabolising the same dietary source—complex carbohydrates (Ormerod et al., 2016; Smith et al., 2019). It is worth considering the extent to which the gut microbiota may be directly utilising zinc from the diet, potentially in competition with the host animal. Smith et al. (1972) estimated that GI bacteria utilise 20% of the dietary zinc consumed in a germ-free/pathogen-free rat study (Smith et al., 1972). Zinc supplementation of the host diet (150 ppm) potentially results in increased zinc availability along the GI tract for the microbial community, affecting its overall community structure and function. Microbial high-affinity membrane transport systems and zinc-binding proteins for competitive uptake of zinc between gut microbes are crucial for their persistence and growth along the GI tract. Balancing host and gut microbiome requirements for zinc is therefore essential for survival of both symbiotic components.

Among the *Shank3B^−/−^* KO mice, the bacterial families *Enterobacteriaceae* and *Coriobacteriaceae* were the top two significant biomarkers that were more abundant in the supplemented zinc diet (KO150) mice compared with the control zinc diet (KO30) mice. KO30 mice, instead, had increased abundances of *Muribaculaceae* and *Ileibacterium*. Diet changes can alter host production of bile acids, which can shift the gut community in favour of organisms that can metabolise bile acids such as members of the *Coriobacteriaceae* (Stenman et al., 2013). This, in turn, can affect gut barrier permeability by interaction of bile acids with the epithelial cells (Raimondi et al., 2008). Similarly, members of *Muribaculaceae*, *Blautia* and *Faecalibaculum* were more abundant in WT30 than KO150 mice, which again had higher abundances of *Coriobacteriaceae* and *Escherichia/Shigella*.

While our main focus was on the influence of dietary zinc on the gut microbiota of *Shank3B^−/−^* KO mice, this study also provided an opportunity to revisit the association between host genotype and microbiota composition in this animal model. An earlier study by Tabouy et al. (2018) described reduced bacterial alpha-diversity in *Shank3B^−/−^* KO compared with wild-type mice, as well as differences in the relative abundances of several bacterial genera including *Lactobacillus* and *Prevotella*. The authors interpreted these differences as reflective of a dysbiosis, or dysregulation, of the microbiome. We also observed differences of a comparable magnitude between the *Shank3B^−/−^* KO and WT mice microbiotas (on the control 30 ppm zinc diet), specifically a decrease in bacterial diversity in KO mice, but contend that these do not necessarily constitute a dysbiosis. Indeed, there were marked similarities at both phylum and genus levels between the *Shank3B^−/−^* KO and wild-type bacterial communities in our data (Figure 1), as well as in the aggregated genus-level data of Tabouy et al. (2018) (Supplementary Figure 2 in their paper). Moreover, while statistically significant, alpha-diversity in the female mice (which displayed the strongest effect) still only decreased by <10% in Tabouy et al. (2018) data set. Widespread use of the term “dysbiosis” to describe any shift in microbial communities has been criticised (Olesen and Alm, 2016) and we suggest that this term would more appropriately apply to a situation of extreme microbiota depletion, such as seen in *Clostridioides difficile* infection (Antharam et al., 2013), rather than the more subtle changes evident in both our data and those of Tabouy et al. (2018). Nonetheless, whether or not the microbiota differences observed here and by Tabouy et al. (2018) constitute a dysbiosis *per se*, the functional implications of reduced gut-associated microbiota diversity in *Shank3B^−/−^* KO mice warrant investigation. It is important to note that genotype and dietary zinc factors explained ~7% of variation in the bacterial biota, compared with almost 13% attributable to individual cage differences. The explanatory power of dietary zinc and genotype was therefore lower, despite striking host behavioural differences associated with zinc supplementation (Fourie et al., 2018), which were not influenced by mouse cage assignment.

While zinc deficiency can be difficult to diagnose in the clinic, plasma serum levels can be influenced by multiple independent factors and use of hair/nail samples have not been conclusive in the literature; therefore proposals for potential microbial biomarkers for zinc deficiency have been suggested (Chen et al., 2021).

Our data also show significant effects of genotype and dietary zinc on fungal diversity. Twenty-four fungal ZOTUs differed significantly in relative abundance across the entire dataset. The five that could be assigned to below kingdom level represented the genera *Rhizopus* and *Aspergillus.* We observed that, on the control diet, KO mice displayed significantly lower fungal diversity compared with WT mice, and moreover that dietary zinc treatment in KO mice reverted fungal diversity towards WT levels. Zinc is key for fungi and has been demonstrated to enhance growth of *Rhizopus* with higher zinc levels (Wegener and Romano, 1963) and *Aspergillus* (Vicentefranqueira et al., 2019) by stimulating RNA synthesis and transcription. Autism-associated behaviours of a child with ASD were reversed following treatment with antifungals to target *Rhizopus*, though the efficacy of this treatment for a wider population is yet to be tested (Baker and Shaw, 2020). Mycotoxins and fungi have been postulated to be involved in the pathobiology of ASD due to their high prevalence in the urine and blood of children on the autism spectrum, but this is largely understudied (De Santis et al., 2019; Markova, 2019). Potential targets for antifungal treatment include zinc transporters (due to their ubiquity and importance in fungi) as well as probiotic treatments such as *Lactobacillus* aimed at adsorbing mycotoxins or altering gut microbial communities (Amich and Calera, 2014; Guo et al., 2014; Vicentefranqueira et al., 2015). Therefore, dietary zinc supplementation may aid in maintaining the balance between a healthy fungal community and its interactions with the *Shank3B*-deficient host which ultimately may affect their phenotype. Together with our data, these findings highlight fungal diversity and zinc as an important focus for future studies.

### 4.2 Dietary zinc supplementation has a minor but measurable effect on host gene expression in the mouse gut

Consideration of host–microbe interactions demands attention to not only the microbiota, but also the host itself. We therefore utilised RNA-Seq to investigate patterns of host (mouse) gene expression in the ileum and colon of both WT and *Shank3B^−/−^* KO mice fed either 30 ppm or 150 ppm dietary zinc. As overall gene expression correlated most strongly with gut region (ileum *vs* colon) rather than experimental group (dietary zinc or genotype) (Figure 5), we focused our attention on those genes that were significantly differentially expressed and/or directly relevant to integrity of the gut epithelial barrier.

Alpha defensins are antimicrobial peptides active against a wide range of microorganisms (De Smet and Contreras, 2005). Consistent with their known prevalence in the small intestine (Takakuwa et al., 2019; Nakamura et al., 2020), we documented higher expression of alpha defensin genes in the ileum compared with colon samples. Expression of several alpha defensins was higher in the *Shank3B^−/−^* KO mice compared to the wild-type mice, especially those on the supplemented zinc diet. Whilst speculative, this could indicate enhanced antimicrobial activity in *Shank3B^−/−^* KO mice, and that this is further enhanced by high dietary zinc. This suggests that the gut microbiome may be more strictly controlled in *Shank3B^−/−^* KO mice compared to wild-type mice by various host–microbe interactions.

Given the significant research focus on potential leaky gut in ASD, we also focused our analysis on the expression of tight junction proteins between WT and *Shank3B^−/−^* KO mice fed control or supplemented zinc levels. Tight junction proteins are crucial for integrity of the gut epithelium, preventing potentially harmful microorganisms or their metabolites from leaking out of the gut and into the bloodstream. Zinc supplementation enhances and maintains the intestinal barrier by regulating expression of several tight junction proteins (Miyoshi et al., 2016). Tight junction expression in the ileum samples from *Shank3B^−/−^* KO mice was higher than in the corresponding WT30 samples, which may suggest the presence of intestinal inflammation or compromised barrier integrity that can ultimately impact the gut-microbiota-brain axis (Berkes et al., 2003). Additionally, *Shank3B^−/−^* KO mice can have abnormal GI morphology, with alterations of the gut microbial community and increased bacterial LPS levels in the liver (Sauer et al., 2019); this suggests the importance of the SHANK3 protein in the gut and the consequences of KO SHANK3 involvement in disruption of the mucosal barrier. Dietary zinc supplementation correlated with higher tight junction gene expression, regardless of host genotype, particularly in the ileum. This is intuitive given the greater absorbance of dietary zinc in the small intestine (Maret, 2000; Dufner-Beattie et al., 2003; Liuzzi et al., 2004). Overall, our observations suggest that zinc may be playing a role in reinforcement of the gut epithelial barrier that may help to tightly regulate the gut-microbiota-brain axis and perhaps ameliorate the resulting ASD behaviours.

### 4.3 Gut region correlates more strongly with fungal than bacterial community composition

We sampled the mouse gut at several regions along the GI tract, namely the ileum, caecum and colon, as well as faecal samples. For bacteria, there was no clear separation of microbiota composition related to gut region in the ordination plot (Figure 3A), although for many individual mice the ileum samples were somewhat distinct. There was much greater separation for fungi, with the fungal composition of faecal samples being markedly different from that of other sample types (Figure 2B) and greater variation explained in the PERMANOVA models compared to the bacterial data. The bacterial community was substantially more diverse than fungal communities across the dataset (Figure 3).

### 4.4 Gut bacterial communities display functional redundancy

Fatty acids produced from microbial fermentation/metabolism, such as butyrate and propionate, modulate gut barrier function and provide an anti-inflammatory effect via inhibition of host immune responses, both of which have been linked to the gut-microbiome-brain axis. In particular, *Roseburia* and *Faecalibaculum* were present across samples and sample groups, and encoded genes for fatty acid biosynthesis, with the greatest relative abundance of *Faecalibaculum* in the KO150 group. However, the main fatty acid producers found in this dataset were *Akkermansia muciniphila, Muribaculum intestinale* and *Alistipes finegoldii*. Therefore, important fatty acid functional genes of the gut microbiome may be expressed by different members of the bacterial community. This suggests that bacterial function rather than identity could be a more important contributor to the reversal of ASD-related behaviours observed in this mouse model.

Overall, a potential reciprocal relationship, with *A. muciniphila* degrading mucin and supplying butyrate to the host, may contribute to the observed effects of supplementary dietary zinc, in concert with direct zinc interactions alongside SHANK3 proteins in brain synapses subsequently supporting the ASD behavioural reversal in *Shank3B^−/^*mice. Understanding crosstalk between a host and its gut microbiota is challenging due to the multitude of communication mechanisms across the gut-microbiota-brain axis, however elucidating specific beneficial functions could aid with GI disorders commonly associated with ASD via probiotic supplementation (Zhang et al., 2022).

## 5 Concluding remarks

To our knowledge this study is the first to investigate the effect of dietary zinc supplementation on the gut microbiota in the context of ASD. By utilising the *Shank3B^−/−^* KO mouse model we were able to examine the influence of – and interaction between – dietary zinc and ASD-linked host genotype. We found that sample location along the GI tract, as well as genotype and zinc diet, explained some of the variation seen in the microbiota data, with notable bacterial ASV differences between experimental groups. While we observed statistically significant differences between the alpha-diversity of microbiotas from different genotype and dietary zinc treatment groups, we argue that these do not necessarily constitute a major breakdown, or dysbiosis, of the gut microbiota. Interestingly, the observed differences in fungal diversity between *Shank3B* KO mice on different zinc diets are consistent with dietary zinc supplementation maintaining a healthy fungal community and potentially influencing ASD phenotype. Differential expression of host genes among the experimental groups, including antimicrobial interaction genes as well as gut microbiota-regulated host metabolism genes, suggests that the interplay between gut microbes, the gastrointestinal tract and the brain may play a major role towards the observed amelioration of ASD behaviours seen previously with supplemented dietary zinc. Future work, perhaps involving targeted manipulation of the identified genes and/or metabolic processes, would represent a valuable next step. We found differences in bacterial functional capacities among our experimental groups, which notably include fatty acid biosynthesis and transporters (including zinc transport and neurotransmitter receptors). These differences could suggest increased activity of, or a switch towards, specific functions that may benefit both host and microbe, raising the potential of manipulating both dietary zinc and the gut microbiota itself to ameliorate ASD-related behaviours and associated GI issues.

## Data Availability

The sequence data from this study have been deposited in the NCBI Sequence Read Archive under BioProject PRJNA747052 (bacterial and fungal amplicon data) and PRJNA764443 (shotgun metagenome and RNA data).
