# Supplementary material for: Effect of dietary zinc supplementation on the gastrointestinal microbiome and host gene expression in the Shank3B−/− mouse model of autism spectrum disorder
Source: Front Microbiol. 2025 Aug 12;16:1607045. doi: 10.3389/fmicb.2025.1607045 (PMC12378474; doi:10.3389/fmicb.2025.1607045)
Supplement: Supplementary file 1 [file Supplementary_file_1.zip › Supplementary Figure 1.docx]

Supplementary Material for:

Effect of dietary zinc supplementation on the gastrointestinal microbiome and host gene expression in the Shank3B-/- mouse model of autism spectrum disorder

Giselle C. Wong, Yewon Jung, Kevin Lee, Chantelle Fourie, Kim M. Handley, Johanna M. Montgomery, Michael W. Taylor


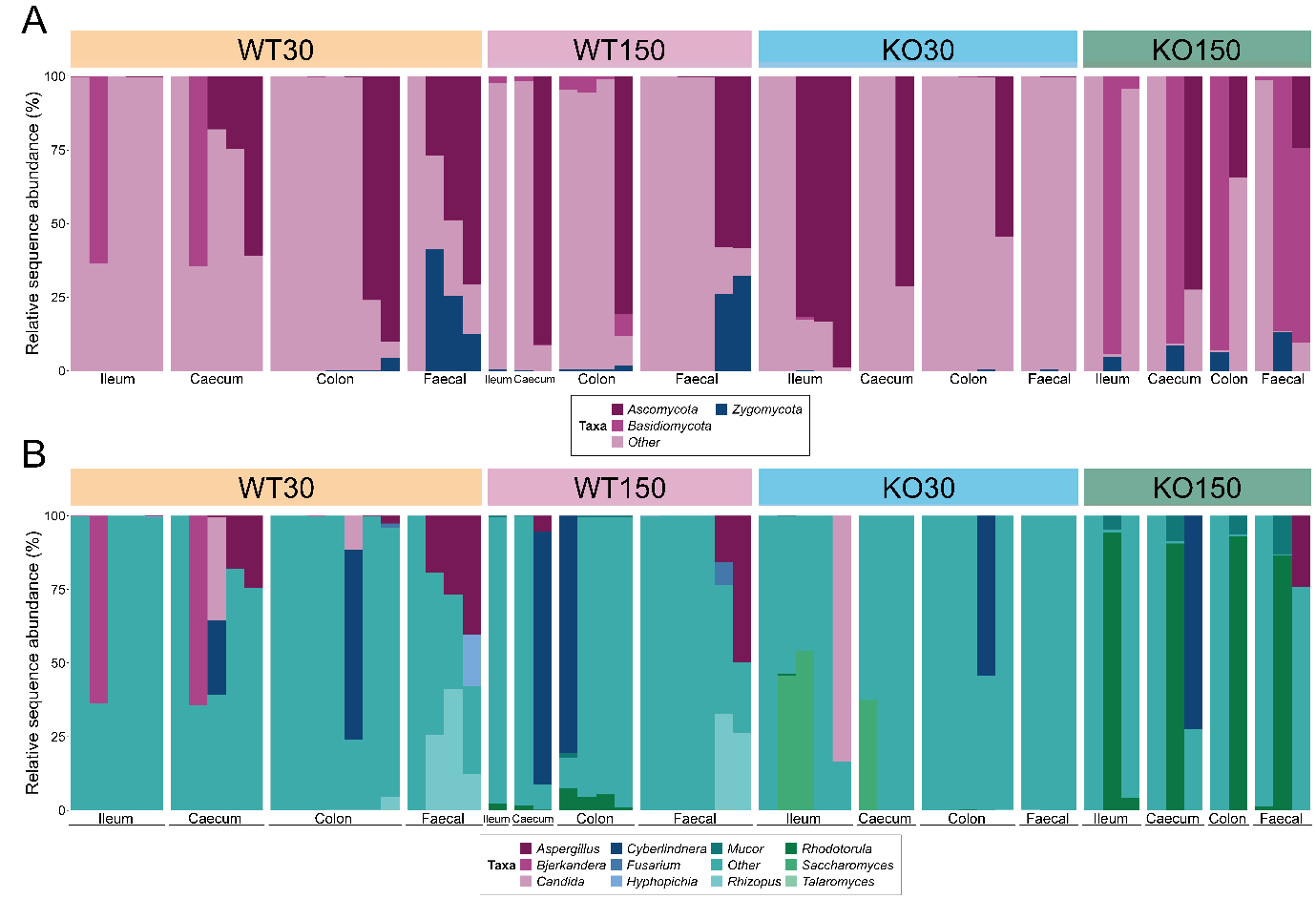


**Figure S1**. ITS2-based taxonomic summary plot showing relative abundance of fungal phyla (A) and the 11 most abundant genera (B) in each of the four experimental groups (wild-type control zinc diet WT30 n = 21, wild-type supplementary zinc diet WT150 n = 13, *Shank3B^-/-^* KO control zinc diet KO30 n = 16, and *Shank3B^-/-^* KO supplementary zinc diet KO150 n = 11). Gastrointestinal sections are noted as I (ileum), Ce (caecum), Co (colon), F (faecal).
